# *N*′-(2-Chloro­benzyl­idene)-2-methyl­benzohydrazide

**DOI:** 10.1107/S1600536812000463

**Published:** 2012-01-11

**Authors:** Wei-Guang Zhang

**Affiliations:** a College of Chemistry and Chemical Engineering, Qiqihar University, Qiqihar 161006, People’s Republic of China

## Abstract

The title hydrazone compound, C_15_H_13_ClN_2_O, adopts an *E* configuration about the C=N double bond. The dihedral angle between the two benzene rings is 13.1 (2)°. In the crystal, mol­ecules are linked through N—H⋯O hydrogen bonds, forming chains parallel to [101].

## Related literature

For the biological properties of hydrazone compounds, see: Ajani *et al.* (2010 ▶); Angelusiu *et al.* (2010 ▶); Zhang *et al.* (2010 ▶); Horiuchi *et al.* (2009 ▶). For the crystal structures of similar hydrazone comounds, see: Ban (2010 ▶); Hussain *et al.* (2010 ▶); Shalash *et al.* (2010 ▶); Khaledi *et al.* (2009 ▶). For the crystal structure of the 2-fluorobenzohydrazide analoque, reported on recently by the author, see: Zhang (2011 ▶).
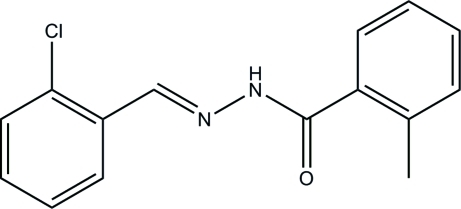


## Experimental

### 

#### Crystal data

C_15_H_13_ClN_2_O
*M* *_r_* = 272.72
Monoclinic, 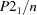

*a* = 7.4305 (17) Å
*b* = 25.596 (2) Å
*c* = 7.7926 (18) Å
β = 113.505 (2)°
*V* = 1359.1 (5) Å^3^
*Z* = 4
Mo *K*α radiation
μ = 0.27 mm^−1^
*T* = 298 K
0.20 × 0.20 × 0.20 mm

#### Data collection

Bruker APEXII CCD area-detector diffractometer
Absorption correction: multi-scan (*SADABS*; Sheldrick, 1996 ▶) *T*_min_ = 0.947, *T*_max_ = 0.947
7513 measured reflections
2516 independent reflections
1870 reflections with *I* > 2σ(*I*)
*R*_int_ = 0.043

#### Refinement

*R*[*F* ^2^ > 2σ(*F* ^2^)] = 0.058
*wR*(*F* ^2^) = 0.141
*S* = 1.08
2516 reflections
176 parameters
1 restraint
H atoms treated by a mixture of independent and constrained refinement
Δρ_max_ = 0.24 e Å^−3^
Δρ_min_ = −0.33 e Å^−3^

### 

Data collection: *APEX2* (Bruker, 2007 ▶); cell refinement: *SAINT* (Bruker, 2007 ▶); data reduction: *SAINT*; program(s) used to solve structure: *SHELXS97* (Sheldrick, 2008 ▶); program(s) used to refine structure: *SHELXL97* (Sheldrick, 2008 ▶); molecular graphics: *SHELXTL* (Sheldrick, 2008 ▶); software used to prepare material for publication: *SHELXTL*.

## Supplementary Material

Crystal structure: contains datablock(s) global, I. DOI: 10.1107/S1600536812000463/qm2047sup1.cif

Structure factors: contains datablock(s) I. DOI: 10.1107/S1600536812000463/qm2047Isup2.hkl

Supplementary material file. DOI: 10.1107/S1600536812000463/qm2047Isup3.cml

Additional supplementary materials:  crystallographic information; 3D view; checkCIF report

## Figures and Tables

**Table 1:** Hydrogen-bond geometry (Å, °)

*D*—H⋯*A*	*D*—H	H⋯*A*	*D*⋯*A*	*D*—H⋯*A*
N2—H2⋯O1^i^	0.90 (1)	2.00 (1)	2.876 (3)	164 (3)

Symmetry code: (i) 
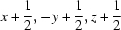
.

**Fig. 1.**
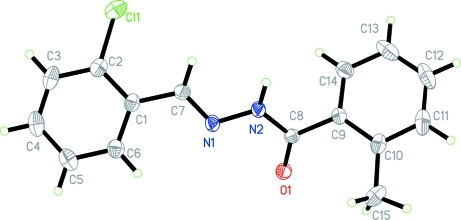
The molecular structure of the title compound, showing the atom-numbering scheme and displacement ellipsoids drawn at the 30% probability level.

**Fig. 2.**
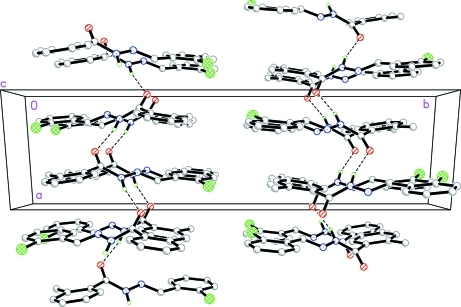
The molecular packing of the title compound viewed along the c axis. Hydrogen bonds are shown as dashed lines. H-atoms not involved in hydrogen bonding have been omitted for clarity.

## supplementary crystallographic information

### Comment

Benzoylhydrazones are a kind of special Schiff bases bearing the –C(O)—NH—N=CH– groups. The hydrazone compounds have been received much attention for their excellent biological properties (Ajani *et al.*, 2010; Angelusiu *et al.*, 2010; Zhang *et al.*, 2010; Horiuchi *et al.*, 2009) as well as their crystal structures (Ban, 2010; Hussain *et al.*, 2010; Shalash *et al.*, 2010; Khaledi *et al.*, 2009). Recently, the author has reported a hydrazone compound derived from the reaction of 2-chlorobenzaldehyde with 2-fluorobenzohydrazide (Zhang, 2011). In the present paper, the title new hydrazone compound, derived from the reaction of 2-chlorobenzaldehyde with 2-methylbenzohydrazide, is reported.

The compound adopts an *E* configuration about the C═N double bond (Fig. 1). The dihedral angle between the two substituted benzene rings is 13.1 (2)°. In the crystal structure, molecules are linked through intermolecular N—H···O hydrogen bonds (Table 1), forming chains parallel to the *ac* diagonal (Fig. 2).

### Experimental

2-Chlorobenzaldehyde (0.140 g, 1 mmol) and 2-methylbenzohydrazide (0.150 g, 1 mmol) were mixed in 50 ml me thanol. The mixture was stirred and refluxed for 30 min and cooled to room temperature to give a colorless solution. Colorless block-shaped single crystals were obtained on slow evaporation of the solution in air.

### Refinement

H2 was located in a difference Fourier map and refined with the N—H distance restrained to 0.90 (1) Å. The remaining H atoms were positioned geometrically, with C—H = 0.93–0.96 Å, and with *U*_iso_(H) = 1.2*U*_eq_(C) and 1.5*U*_eq_(C15).

### Crystal data

**Table:**

C_15_H_13_ClN_2_O	*F*(000) = 568
*M**_r_* = 272.72	*D*_x_ = 1.333 Mg m^−^^3^
Monoclinic, *P*2_1_/*n*	Mo *K*α radiation, λ = 0.71073 Å
*a* = 7.4305 (17) Å	Cell parameters from 2213 reflections
*b* = 25.596 (2) Å	θ = 2.7–24.5°
*c* = 7.7926 (18) Å	µ = 0.27 mm^−^^1^
β = 113.505 (2)°	*T* = 298 K
*V* = 1359.1 (5) Å^3^	Block, colorless
*Z* = 4	0.20 × 0.20 × 0.20 mm

### Data collection

**Table:**

Bruker APEXII CCD area-detector diffractometer	2516 independent reflections
Radiation source: fine-focus sealed tube	1870 reflections with *I* > 2σ(*I*)
graphite	*R*_int_ = 0.043
ω scans	θ_max_ = 25.5°, θ_min_ = 3.0°
Absorption correction: multi-scan (*SADABS*; Sheldrick, 1996)	*h* = −8→7
*T*_min_ = 0.947, *T*_max_ = 0.947	*k* = −25→31
7513 measured reflections	*l* = −9→9

### Refinement

**Table:**

Refinement on *F*^2^	Primary atom site location: structure-invariant direct methods
Least-squares matrix: full	Secondary atom site location: difference Fourier map
*R*[*F*^2^ > 2σ(*F*^2^)] = 0.058	Hydrogen site location: inferred from neighbouring sites
*wR*(*F*^2^) = 0.141	H atoms treated by a mixture of independent and constrained refinement
*S* = 1.08	*w* = 1/[σ^2^(*F*_o_^2^) + (0.0445*P*)^2^ + 1.1147*P*] where *P* = (*F*_o_^2^ + 2*F*_c_^2^)/3
2516 reflections	(Δ/σ)_max_ < 0.001
176 parameters	Δρ_max_ = 0.24 e Å^−^^3^
1 restraint	Δρ_min_ = −0.33 e Å^−^^3^

### Special details

**Table:**

Geometry. All e.s.d.'s (except the e.s.d. in the dihedral angle between two l.s. planes) are estimated using the full covariance matrix. The cell e.s.d.'s are taken into account individually in the estimation of e.s.d.'s in distances, angles and torsion angles; correlations between e.s.d.'s in cell parameters are only used when they are defined by crystal symmetry. An approximate (isotropic) treatment of cell e.s.d.'s is used for estimating e.s.d.'s involving l.s. planes.
Refinement. Refinement of *F*^2^ against ALL reflections. The weighted *R*-factor *wR* and goodness of fit *S* are based on *F*^2^, conventional *R*-factors *R* are based on *F*, with *F* set to zero for negative *F*^2^. The threshold expression of *F*^2^ > σ(*F*^2^) is used only for calculating *R*-factors(gt) *etc*. and is not relevant to the choice of reflections for refinement. *R*-factors based on *F*^2^ are statistically about twice as large as those based on *F*, and *R*- factors based on ALL data will be even larger.

### Fractional atomic coordinates and isotropic or equivalent isotropic displacement parameters (Å^2^)

**Table:**

	*x*	*y*	*z*	*U*_iso_*/*U*_eq_	
Cl1	0.29514 (17)	0.04920 (3)	0.54672 (14)	0.0790 (4)	
N1	0.1795 (3)	0.21108 (8)	0.4023 (3)	0.0380 (5)	
N2	0.2213 (3)	0.24341 (8)	0.5565 (3)	0.0388 (6)	
O1	0.0050 (3)	0.30528 (7)	0.3860 (2)	0.0468 (5)	
C1	0.2276 (4)	0.12767 (10)	0.2918 (4)	0.0374 (6)	
C2	0.2488 (4)	0.07402 (11)	0.3250 (4)	0.0465 (7)	
C3	0.2323 (5)	0.03896 (12)	0.1830 (5)	0.0581 (9)	
H3	0.2448	0.0033	0.2069	0.070*	
C4	0.1976 (5)	0.05753 (14)	0.0072 (5)	0.0611 (9)	
H4	0.1865	0.0343	−0.0882	0.073*	
C5	0.1791 (5)	0.11049 (13)	−0.0288 (4)	0.0546 (8)	
H5	0.1573	0.1229	−0.1476	0.066*	
C6	0.1931 (4)	0.14482 (11)	0.1120 (4)	0.0442 (7)	
H6	0.1792	0.1804	0.0863	0.053*	
C7	0.2488 (4)	0.16495 (10)	0.4419 (4)	0.0386 (6)	
H7	0.3131	0.1549	0.5664	0.046*	
C8	0.1263 (4)	0.28972 (10)	0.5363 (3)	0.0331 (6)	
C9	0.1778 (4)	0.31909 (10)	0.7153 (4)	0.0349 (6)	
C10	0.2087 (4)	0.37314 (11)	0.7235 (4)	0.0432 (7)	
C11	0.2488 (5)	0.39768 (14)	0.8949 (5)	0.0634 (10)	
H11	0.2718	0.4335	0.9046	0.076*	
C12	0.2553 (5)	0.37064 (17)	1.0496 (5)	0.0728 (11)	
H12	0.2811	0.3883	1.1612	0.087*	
C13	0.2242 (5)	0.31800 (17)	1.0403 (4)	0.0663 (10)	
H13	0.2282	0.2997	1.1450	0.080*	
C14	0.1869 (4)	0.29217 (12)	0.8746 (4)	0.0475 (7)	
H14	0.1673	0.2562	0.8686	0.057*	
C15	0.2020 (5)	0.40465 (12)	0.5579 (5)	0.0598 (9)	
H15A	0.2780	0.3874	0.4998	0.090*	
H15B	0.2554	0.4388	0.5990	0.090*	
H15C	0.0685	0.4078	0.4693	0.090*	
H2	0.312 (4)	0.2342 (13)	0.669 (3)	0.080*	

### Atomic displacement parameters (Å^2^)

**Table:**

	*U*^11^	*U*^22^	*U*^33^	*U*^12^	*U*^13^	*U*^23^
Cl1	0.1180 (9)	0.0447 (5)	0.0724 (6)	0.0103 (5)	0.0360 (6)	0.0145 (4)
N1	0.0397 (13)	0.0355 (12)	0.0320 (11)	0.0014 (10)	0.0069 (10)	−0.0057 (9)
N2	0.0427 (14)	0.0341 (12)	0.0288 (11)	0.0062 (10)	0.0031 (10)	−0.0042 (9)
O1	0.0516 (12)	0.0383 (10)	0.0342 (10)	0.0068 (9)	−0.0002 (9)	−0.0008 (8)
C1	0.0312 (14)	0.0343 (14)	0.0432 (15)	−0.0029 (11)	0.0111 (12)	−0.0065 (12)
C2	0.0450 (18)	0.0389 (16)	0.0532 (18)	0.0018 (13)	0.0172 (14)	−0.0017 (13)
C3	0.058 (2)	0.0368 (17)	0.082 (2)	−0.0037 (14)	0.0298 (19)	−0.0161 (16)
C4	0.060 (2)	0.061 (2)	0.066 (2)	−0.0093 (17)	0.0283 (18)	−0.0288 (18)
C5	0.0511 (19)	0.070 (2)	0.0457 (17)	−0.0116 (16)	0.0223 (15)	−0.0140 (15)
C6	0.0418 (16)	0.0439 (16)	0.0436 (16)	−0.0045 (13)	0.0134 (13)	−0.0057 (13)
C7	0.0382 (16)	0.0374 (15)	0.0344 (14)	0.0021 (12)	0.0084 (12)	0.0002 (12)
C8	0.0316 (14)	0.0319 (13)	0.0313 (13)	−0.0028 (11)	0.0078 (11)	0.0004 (11)
C9	0.0266 (14)	0.0389 (15)	0.0357 (14)	0.0043 (11)	0.0087 (11)	−0.0038 (11)
C10	0.0307 (15)	0.0389 (15)	0.0531 (17)	0.0001 (12)	0.0096 (13)	−0.0090 (13)
C11	0.050 (2)	0.054 (2)	0.074 (2)	0.0003 (15)	0.0113 (18)	−0.0291 (18)
C12	0.062 (2)	0.097 (3)	0.0458 (19)	0.013 (2)	0.0072 (17)	−0.033 (2)
C13	0.057 (2)	0.103 (3)	0.0362 (17)	0.022 (2)	0.0159 (15)	−0.0005 (18)
C14	0.0462 (18)	0.0549 (18)	0.0384 (15)	0.0074 (14)	0.0137 (13)	0.0003 (13)
C15	0.054 (2)	0.0391 (17)	0.085 (2)	−0.0032 (15)	0.0269 (18)	0.0055 (16)

### Geometric parameters (Å, °)

**Table:**

Cl1—C2	1.742 (3)	C7—H7	0.9300
N1—C7	1.276 (3)	C8—C9	1.494 (3)
N1—N2	1.388 (3)	C9—C14	1.397 (4)
N2—C8	1.356 (3)	C9—C10	1.400 (4)
N2—H2	0.899 (10)	C10—C11	1.396 (4)
O1—C8	1.225 (3)	C10—C15	1.505 (4)
C1—C6	1.391 (4)	C11—C12	1.374 (5)
C1—C2	1.395 (4)	C11—H11	0.9300
C1—C7	1.468 (4)	C12—C13	1.364 (5)
C2—C3	1.392 (4)	C12—H12	0.9300
C3—C4	1.374 (5)	C13—C14	1.377 (4)
C3—H3	0.9300	C13—H13	0.9300
C4—C5	1.380 (5)	C14—H14	0.9300
C4—H4	0.9300	C15—H15A	0.9600
C5—C6	1.377 (4)	C15—H15B	0.9600
C5—H5	0.9300	C15—H15C	0.9600
C6—H6	0.9300		
			
C7—N1—N2	114.4 (2)	O1—C8—C9	123.1 (2)
C8—N2—N1	119.6 (2)	N2—C8—C9	113.8 (2)
C8—N2—H2	120 (2)	C14—C9—C10	119.9 (3)
N1—N2—H2	121 (2)	C14—C9—C8	119.0 (2)
C6—C1—C2	117.3 (2)	C10—C9—C8	121.0 (2)
C6—C1—C7	121.0 (2)	C11—C10—C9	117.2 (3)
C2—C1—C7	121.6 (2)	C11—C10—C15	120.0 (3)
C3—C2—C1	121.3 (3)	C9—C10—C15	122.8 (3)
C3—C2—Cl1	118.3 (2)	C12—C11—C10	122.1 (3)
C1—C2—Cl1	120.4 (2)	C12—C11—H11	118.9
C4—C3—C2	119.5 (3)	C10—C11—H11	118.9
C4—C3—H3	120.3	C13—C12—C11	120.3 (3)
C2—C3—H3	120.3	C13—C12—H12	119.8
C3—C4—C5	120.4 (3)	C11—C12—H12	119.8
C3—C4—H4	119.8	C12—C13—C14	119.4 (3)
C5—C4—H4	119.8	C12—C13—H13	120.3
C6—C5—C4	119.7 (3)	C14—C13—H13	120.3
C6—C5—H5	120.2	C13—C14—C9	121.1 (3)
C4—C5—H5	120.2	C13—C14—H14	119.4
C5—C6—C1	121.7 (3)	C9—C14—H14	119.4
C5—C6—H6	119.1	C10—C15—H15A	109.5
C1—C6—H6	119.1	C10—C15—H15B	109.5
N1—C7—C1	120.3 (2)	H15A—C15—H15B	109.5
N1—C7—H7	119.9	C10—C15—H15C	109.5
C1—C7—H7	119.9	H15A—C15—H15C	109.5
O1—C8—N2	123.1 (2)	H15B—C15—H15C	109.5

### Hydrogen-bond geometry (Å, °)

**Table:**

*D*—H···*A*	*D*—H	H···*A*	*D*···*A*	*D*—H···*A*
N2—H2···O1^i^	0.90 (1)	2.00 (1)	2.876 (3)	164 (3)

Symmetry codes: (i) *x*+1/2, −*y*+1/2, *z*+1/2.
